# Uric Acid

**Published:** 1898-03-26

**Authors:** 


### URIC ACID.

The question whether uric acid compounds exist in healthy blood continues to excite considerable interest among the more chemically-minded in our ranks, while even clinicians, satisfied as they may feel as to the benefits derived from the use of alkalies and salicylates in gouty ailments, would be very glad to have some reasonable grounds for their beliefs.

The matter came up again at the meeting of the Royal Medical and Chirurgical Society held last Tuesday, when Dr. Haig gave a demonstration of Mr. Barker-Smith's chloride of ammonium process for the detection of uric acid in the blood.

The process is comparatively simple, and, if reliable, will be of very great service in the daily work of the physician. The directions are as follows: Place a minute drop of blood on a microscope slide, mix with a similar quantity of a 10 per cent. solution of carbonate of sodium, and then with a similar quantity of a 20 per cent. solution of chloride of ammonium, and put on a cover glass. Allow to stand for 30 minutes, preventing evaporation. Examine under a one-sixth inch or one-eighth inch objective. Pale granules, generally spherical, and about one-sixth to one-third the size of a red blood-cell, are seen all over the field. The author thought that by counting all the granules in a field, and then all the red cells in the same field, a quantitive relation was obtained which might be of some value. At any rate, the number of these granules in blood thus treated appeared to correspond in a remarkable way with the amount of uric acid passing in the urine, and it was noteworthy that their number also corresponded with what was known about uric acid in the blood of disease, the granules being very numerous in Bright's disease, especially in the large pale kidney, and in chronic gout, and very scanty in early and acute fever.

Dr. Luff, however, traversed all this, maintaining that, before these granules could be taken as a guide to the existence of uric acid, they should be shown to contain it or its derivatives, a thing of which no proof whatever had hitherto been offered. He moreover asserted that, as a fact, uric acid did not exist in healthy blood, and, therefore, that a process which showed a reaction with such blood must be a fallacious guide to the presence of uric acid, and he described how he had obtained the same reaction from a solution of cheese, which he supposed even Dr. Haig would admit to be free from uric acid.

Dr. Haig, however, was not to be shaken by trifles such as these, and he challenged Dr. Luff to test the method experimentally, offering himself to examine the blood of a patient, half-hour by half-hour, and to plot out the rise and fall of uric acid as shown by the "granules," if Dr. Haig would, by chemical examination of the urine, at the same time plot out the actual amount of uric acid compounds passing out at corresponding periods by the kidneys. Both tables could then be shown to the president, who would find that they both told the same tale. Whether the society thought that this offer savoured too much of sport or of frivolity we cannot say, but it passed on to the next subject. We cannot, however, forbear to remark that it is somewhat of a reproach that at this time of day men of light and leading should still remain in opposite camps regarding a subject which surely ought to be capable of a positive answer one way or another. If science cannot tell us, so positively than none can disbelieve, whether or no uric acid exists in healthy blood, what faith are we to place in all the peculiarly minute and elaborate analyses of the various secretions of the body which physiological chemists offer from time to time for our admiration and wonderment.

